# Combining individual *Chlamydia trachomatis* IgG antibodies MOMP, TARP, CPAF, OMP2, and HSP60 for tubal factor infertility prediction

**DOI:** 10.1111/aji.13091

**Published:** 2019-02-06

**Authors:** Eleanne F. van Ess, Anat Eck-Hauer, Jolande A. Land, Servaas A. Morré, Sander Ouburg

**Affiliations:** ^1^ Department of Medical Microbiology & Infection Control, Laboratory of Immunogenetics, Amsterdam UMC VU University Medical Centre Amsterdam The Netherlands; ^2^ Department of Genetics and Cell Biology, Faculty of Health, Medicine & Life Sciences, Institute for Public Health Genomics (IPHG), Research Institute GROW University of Maastricht Maastricht The Netherlands

**Keywords:** *Chlamydia trachomatis*, IgG, immunoblot, immunology, prediction, serology, tubal factor infertility, tubal infertility

## Abstract

**Problem:**

Tubal factor infertility (TFI) is a severe complication of genital *Chlamydia trachomatis* infections. In fertility workup, chlamydia antibody test (CAT) is used to predict TFI. The predictive value for TFI of most commonly used CAT is moderate.

**Method of study:**

A total of 183 infertile Dutch Caucasian women were included in this study. All underwent tubal patency testing (hysterosalpingography [HSG] or laparoscopy). Cases had TFI, and controls had no TFI (ie normal findings during HSG or laparoscopy). TFI was categorized based on severity (TFI 1‐TFI 4). This study investigated the predictive values of major outer membrane protein (MOMP), translocated actin‐recruiting phosphoprotein (TARP), chlamydial protease‐like activity factor (CPAF), heat shock protein‐60 (HSP60) and outer membrane protein 2 (OMP2) for TFI. A predictive algorithm is developed to detect TFI with a high certainty based on combinations of antibody titres. Serum was tested with the Mikrogen recomLine immunoblot and quantified with the recomScan. A greedy algorithm that explores all possible antibody combinations was developed.

**Results:**

Significant differences in the distributions of antigen titres between cases and controls were observed for CPAF (*P* = 0.0021), HSP60 (*P* = 0.0061), MOMP (*P* = 0.0497) and OMP2 (*P* = 0.0016). Single antibodies could not discriminate between TFI and controls by themselves. The greedy algorithm performs better in specificity, positive predictive value (PPV), accuracy and clinical utility index than the original Mikrogen algorithm. CPAF combined with HSP60 identified 18.2% of TFI cases with 100% certainty. Most of the TFI 4 cases were identified with cut‐offs of CPAF > 10.7 or OMP2 > 3.9.

**Conclusion:**

This proof‐of‐principle study shows that combinations of antibodies in serum are predictive for TFI. A commercially available test can be adapted to predict TFI with a 100% specificity.

## 1 INTRODUCTION

*Chlamydia trachomatis* (*C* *trachomatis*) infections are the most prevalent bacterial sexually transmitted infections (STI) worldwide: WHO estimated 130.9 million new cases in 2012.^1^ The health burden of *C* *trachomatis* infections is high due to its asymptomatic course in up to 80% of women and 50% of men.^2^, ^3^ In women, unnoticed and thus untreated urogenital *C* *trachomatis* infections can lead to severe complications such as pelvic inflammatory disease (PID), ectopic pregnancy and tubal factor infertility (TFI).

*Chlamydia trachomatis* infections are most prevalent in young adolescents, but complications such as TFI can become evident more than 10 years later when these women fail to conceive and present with infertility. By that time, the *C* *trachomatis* bacterium has been cleared by the immune system, and *C* *trachomatis* DNA is not detectable any more by PCR. Serum *C* *trachomatis* IgG antibodies however may remain detectable for many years after infection, even after antibiotic treatment.^4^

In Dutch fertility clinics, serological chlamydia antibody tests (CAT) are used in infertile women as markers of a previous *C* *trachomatis* infection and to estimate the risk of TFI. Based on CAT, high‐risk patients for TFI might be referred for invasive diagnostic testing (eg laparoscopy), whereas in low‐risk patients, less‐invasive procedures (eg hysterosalpingography) might be preferred or it may be decided to refrain from further testing.

The predictive value for TFI of the currently most frequently used CAT is poor, with a negative predictive value (NPV) of 74%‐90% and a positive predictive value (PPV) for TFI of 32%‐63%.^5^, ^6^, ^7^ These poor predictive values of the CAT are due to the fact that CATs are designed to detect *C* *trachomatis* antibodies and not TFI. In clinical practice, false‐positive CAT results may lead to unnecessary laparoscopies in women without TFI, and false‐negative CAT results may cause delay in diagnosis and treatment in women with TFI.

The CAT that is most frequently used in Dutch fertility clinics is the mono‐target Medac *C* *trachomatis* IgG ELISA plus, which is a MOMP‐peptide‐based assay. This enzyme‐linked immunosorbent assay (ELISA) detects antibodies against het major outer membrane protein (MOMP) on the *C* *trachomatis* cell surface and is considered as species specific with a minimal cross‐reactivity with *Chlamydia pneumoniae* antibodies.^5^ MOMP is an immunodominant protein that is involved in maintaining rigidity of the chlamydial membrane, attachment to the human epithelial cell and functions as a pore to provide the bacterium with nutrients once it has invaded the human cell.^8^, ^9^, ^10^ A previous study compared the predictive value for TFI of the Medac ELISA *plus* (Medac GmbH, Wedel, Germany) with a multi‐target Mikrogen ELISA and immunoblot.^7^ This multi‐target ELISA detects antibodies directed against MOMP, translocated actin‐recruiting phosphoprotein (TARP; involved in the internalization of *Chlamydia* into the host cell) and chlamydial protease‐like activity factor (CPAF; involved in host and bacterial protein regulation and bacterial survival), all immunodominant *C* *trachomatis* epitopes. Even though the multi‐target ELISA detects a broader spectrum of *C* *trachomatis* IgG antibodies, no significant improvement of the predictive value for TFI was found.^7^ The disadvantage of a multi‐target ELISA is that it does not allow differentiation of *C* *trachomatis* IgG antibodies against different antigens, and thus, it remains unclear which antibodies are positive in one well. It is hypothesized that certain *C* *trachomatis* IgG antibodies are more predictive for TFI than others. For example, chlamydial heat shock protein‐60 (HSP60) and CPAF have been found to be more prevalent in women with TFI as compared to fertile women.^6^, ^11^, ^12^ Therefore, analysing the presence and composition of individual antibodies in infertile women is of great importance.

The Mikrogen immunoblot detects *C* *trachomatis* IgG antibodies directed against MOMP, TARP, CPAF, cHSP60 and outer membrane protein 2 (OMP2), which are immunodominant *C* *trachomatis* proteins. The benefit of an immunoblot is that it allows differentiation between the *C* *trachomatis* IgG antibodies in the serum. Although previous research showed that ELISAs have a higher sensitivity than immunoblots, the specificity of the immunoblot is higher than the ELISA.^13^, ^14^ However, there is no significant difference in the NPV and PPV of the Mikrogen *C* *trachomatis* IgG ELISA and immunoblot.^15^ Therefore, it is interesting to analyse the predictive value for TFI of the separate antibodies in the Mikrogen immunoblot and of different antibody titre cut‐off values and to adjust the algorithm of the immunoblot analysing software in order to improve the prediction of TFI.

There is a clinical unmet need for improvement of the clinical predictive value of CAT for TFI, since CAT assays have been validated in STI patients and not TFI patients specifically. In this proof‐of‐principle study, we research the contribution of separate types of antibodies in the prediction of TFI, in order to create a predictive value of CAT for TFI. We will also research whether adjustments in cut‐off values will increase the clinical predictive value of *C* *trachomatis* serology for TFI. Since previous studies have shown that different serological tests do not lead to an increase in PPV and NPV for TFI, our aim is to find a combination and cut‐off of antibody titres that detects the most TFI cases with a very high certainty.

## 2 METHODS

### 2.1 Sample collection and definitions

**Figure 1:**
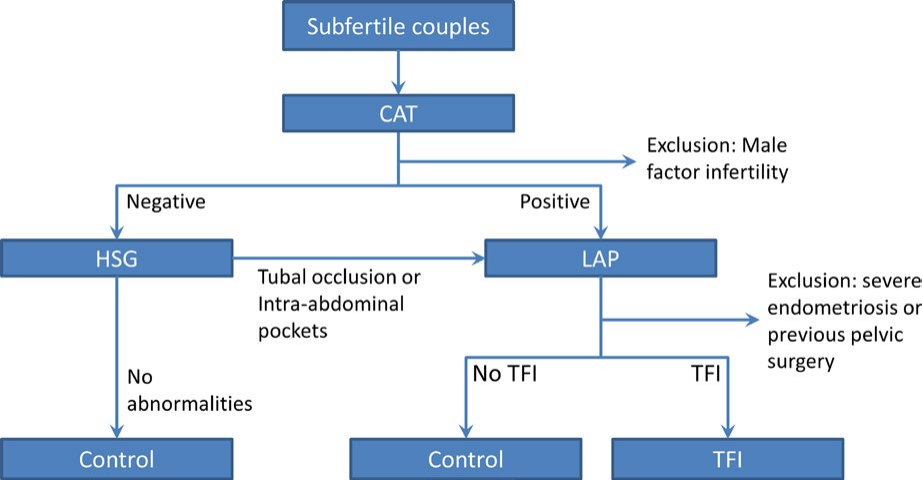
Flow diagram of the inclusion of patients in the study. CAT, chlamydia antibody test; HSG, hysterosalpingography; LAP, laparoscopy; TFI, tubal factor infertility

The study was performed in Dutch Caucasian women, between the age of 18 and 41, who visited the fertility clinic of the University Medical Center Groningen (UMCG) between 2007 and 2013 because of infertility (ie not having conceived after at least 1 year of unprotected intercourse). As part of the fertility workup, blood was drawn in all women and CAT (Medac ELISA plus) was determined. All spare sera were cryopreserved in −20°C. After excluding couples with severe male factor infertility, CAT‐positive women were referred for laparoscopy with tubal testing. In CAT‐negative women, hysterosalpingography (HSG) was performed, and when tubal occlusion or intra‐abdominal pockets were seen on HSG, patients were referred for laparoscopy to verify the presence or absence of TFI. In women with bilateral patent tubes on HSG, no additional testing was done because of the high NPV of HSG.^16^ Only women with available CAT results and who had undergone HSG and/or laparoscopy were included in the present study. Patients who were diagnosed with severe endometriosis on laparoscopy and patients who had undergone previous pelvic surgery (except for an uneventful appendectomy or Caesarean section) were excluded from this study. The inclusion is graphically represented by the flow diagram in Figure 1. No data had been systematically collected on previous sexually transmitted diseases and *C* *trachomatis* infections.

For this study, 158 patients were selected from 613 consecutive subfertile patients from the UMCG fertility clinic between 2007 and 2013. These 158 patients consist of all CAT positives and all TFI‐positive women, and women with a negative CAT, women with negative TFI and women without abnormal HSG. We chose a distribution of 1:2.5 for cases and controls.

Tubal factor infertility was categorized into TFI 1, TFI 2, TFI 3 and TFI 4, respectively, based on different definitions representing the degree and location of abnormalities.^17^ In TFI 1, tubal pathology was defined as any peritubal and/or periovarian adhesions, and/or proximal or distal occlusion of at least one tube, and in TFI 2, tubal pathology was defined as extensive periadnexal adhesions and/or proximal occlusion of at least one tube. In TFI 3, tubal pathology was defined as extensive periadnexal adhesions and/or distal occlusion of one tube, and in TFI 4, tubal pathology was defined as extensive periadnexal adhesions and/or distal occlusion of both tubes. TFI 3 and TFI 4 are considered to represent severe TFI, and these women have a very limited or no chance to conceive naturally.

Controls had no abnormalities on HSG and/or did not fulfil any definition of TFI at laparoscopy.

Of the 158 women included in this study, 33 (20.9%) had TFI. The others were defined as controls. Within the TFI groups, no patients had TFI 1, 11 patients had TFI 2, 14 patients had TFI 3, and 8 patients had TFI 4.

### 2.2 Serological methods

For analysis of the individual antibodies in the serum samples, the Mikrogen recomLine Chlamydia IgG immunoblot was used (Mikrogen GmbH, Neuried, Germany). The Mikrogen recomLine immunoblot is a nitrocellulose strip immunoassay with recombinant species‐specific antigens and detects IgG antibodies against *C* *trachomatis*, *C* *psittaci* and *C* *pneumoniae*. For this study, only antibodies directed against *C* *trachomatis* antigens MOMP, TARP, CPAF, cHSP60 and OMP2 were taken into account. Workup of the cryopreserved blood samples was according to the manufacturer's instructions.

The immunoassay strips were analysed with the “recomScan” software from Mikrogen, which classifies samples as positive, borderline or negative based on an algorithm of summation of the individual antibodies that are present in a blood sample.^18^

### 2.3 Ethical approval

Women attending the UMCG fertility clinic between 2007 and 2013 were offered a broad “no objection” procedure. The participating women declared no objection for the use of their anonymized medical data and spare serum samples. This study was approved by the medical ethical board of the VU University Medical Center in Amsterdam.

### 2.4 Statistical analyses

Frequencies of TFI patients identified with the decision rules are presented in Table 1. The Wilcoxon‐Mann‐Whitney test was used to assess the mean difference in the distribution of the individual antigen titres between TFI patients and controls. A *P*‐value < 0.05 was considered significant. Corrections for multiple testing were done with the Holm‐Bonferroni test. Analyses were done using R version 3.4.4.

**Table 1:** Comparison of “sensitivity,” “specificity,” “predictive values,” clinical “accuracy” and clinical utility indices (CUI) for the immunoblot with positivity defined according to the manufacturer's instructions and according to our greedy algorithm^19^

Cut‐off false positives	TFI identified	“False positives” (%)	Sens	Spec	PPV	NPV	Accuracy	CUI+	CUI−	Decision rule
0.00	6	0 (0.0)	18.2	100.0	100.0	82.2	82.9	18.2	82.2	CPAF > 9.5 or HSP60 > 3.9
0.05	6	0 (0.0)	18.2	100.0	100.0	82.2	82.9	18.2	82.2	CPAF > 9.5 or HSP60 > 3.9
0.10	6	0 (0.0)	18.2	100.0	100.0	82.2	82.9	18.2	82.2	CPAF > 9.5 or HSP60 > 3.9
0.15	9	1 (10.0)	27. 3	99.2	90.0	83. 8	84.2	24.5	83.1	CPAF > 8.8 or HSP60 > 3.9
0.20	10	2 (16.7)	30.3	98.4	83.3	84.2	84.2	25.3	82.9	CPAF > 8.8 & MOMP > 8.1, or OMP2 > 4.3
0.25	10	2 (16.7)	30.3	98.4	83.3	84.2	84.2	25.3	82.9	CPAF > 8.8 & MOMP > 8.1, or OMP2 > 4.3
0.30	11	3 (21.4)	33.3	97.6	78.6	84.7	84.2	26.2	82.7	CPAF > 10.7 or OMP2 > 3.9
0.35	11	3 (21.4)	33.3	97.6	78.6	84.7	84.2	26.2	82.7	CPAF > 10.7 or OMP2 > 3.9
0.40	11	3 (21.4)	33.3	97.6	78.6	84.7	84.2	26.2	82.7	CPAF > 10.7 or OMP2 > 3.9
Immunoblot original results	19	34 (64.2)	57.6	72.8	35.9	86.7	69.6	20.6	63.1	
Total TFI	33									

CPAF, chlamydial protease‐like activity factor; HSP60, heat shock protein‐60; MOMP, major outer membrane protein; NPV, negative predictive value; OMP2, outer membrane protein 2; PPV, positive predictive value; TFI, tubal factor infertility.

NB: The immunoblot was never designed to primarily detect TFI.

### 2.5 Algorithm for an optimal prediction rule

In order to identify the optimal prediction rule to discriminate TFI patients from controls, we developed a greedy algorithm that explores the set of all possible combinations of parameters (MOMP, TARP, CPAF, HSP60 and OMP2). Within each subset of parameters, the algorithm checks for all possible combinations of logical operators (ie “and” and “or”). The number of subsets of a given size *k *in a set of *n *elements is given by $\left(\begin{array}{c} n\\ k \end{array}\right)$. A subset of size *k* requires *k *− 1 logical operators, and the number of possible combinations is 2*^k^* ^−1^. The total number of combinations tested is therefore given by

$$\left|S\right|=\sum_{k=1}^{n}\left(\begin{array}{c} n\\ k \end{array}\right)2^{k-1}$$

In our case, *n* = 5 and the total number of combinations tested is 3165.

For each subset with specific logical operators, we searched for an optimal cut‐off for each parameter. The search was done based on the empirical values measured for each parameter. The resulting prediction rule includes the parameters to include, their corresponding cut‐offs, and the logical operators to bind them together.

A greedy algorithm keeps the best solution after each iteration. Therefore, it is usually computationally intensive and often requires heuristics. In our case, since there was only a small number of parameters (*n* = 5), we were able to calculate all the possible options. The optimal prediction rule was defined as the rule that could maximize the number of true positives with an additional constraint such that the ratio of false to true positives detected does not exceed 20%. The algorithm was performed using R version 3.4.4.

## 3 RESULTS

### 3.1 Individual antigens

**Figure 2:**
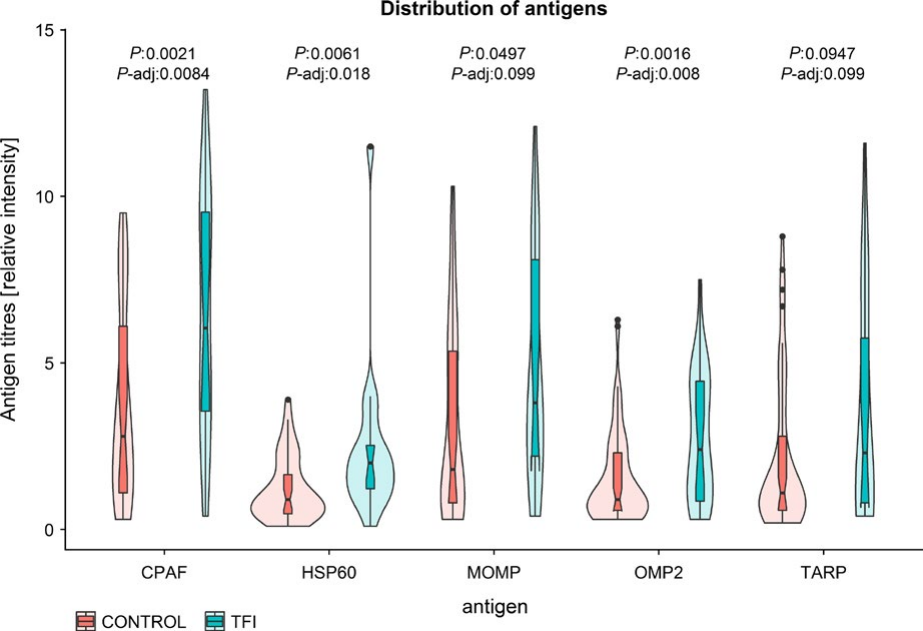
Distribution of the antigens CPAF, HSP60, MOMP, OMP2 and TARP in TFI patients (blue plots) and TFI‐negative controls (red plots). Violin plots show the distribution of the antigen titres. The width of the violin represents the number of samples for the particular titres. The heights of the violin represent the height of the titre. Antigen titres are measured as the relative intensity of the bands on the immunoblot by the recomScan. Boxplots in the “violins” show the median, interquartile range and outliers. Notches in the boxplots show the confidence intervals around the median. Wilcoxon‐Mann‐Whitney *P*‐values and Holm‐Bonferroni‐adjusted *P*‐values are given for each antigen. CPAF, chlamydial protease‐like activity factor; HSP60, heat shock protein‐60; MOMP, major outer membrane protein; OMP2, outer membrane protein 2; TARP, translocated actin‐recruiting phosphoprotein; TFI, tubal factor infertility

The distribution of the CPAF, HSP60, MOMP, OMP2 and TARP antigens as observed in this study is presented in Figure 2. Significant differences in the distributions of antigen titres between TFI patients and controls were observed for CPAF (*P* = 0.0021), HSP60 (*P* = 0.0061), MOMP (*P* = 0.0497) and OMP2 (*P* = 0.0016). After Holm‐Bonferroni correction for multiple testing, CPAF (*P*_adj._ = 0.0084), HSP60 (*P*_adj._ = 0.018) and OMP2 (*P*_adj._ = 0.008) remained significant. As can be seen in Figure 2, due to the overlap in antigen titres, none of the individual antigens can distinguish between TFI patients and controls by itself.

### 3.2 Combined antigens

We employed a greedy algorithm to create a prediction rule to distinguish between TFI cases and controls. The algorithm allows to define what percentage of “false positives” (ie non‐TFI patients with positive test results) will be included. In the algorithm, this percentage is defined as the maximum percentage of identified “true positives” (ie TFI patients with positive test results) that may be “false positives,” for example if the “false positive” cut‐off is 20% and the algorithm identifies 10 TFI cases, then no more than 2 “false positives” are allowed (Table 1).

By allowing the algorithm to include more non‐TFI patients (“false positives”), the algorithm is able to include more true TFI patients resulting in almost doubling of the sensitivity, with only a slight reduction in specificity. Slight increases were observed in the NPV, the accuracy and the clinical utility indices (Table 1). Change in the maximum allowed percentage of “false positives” also changes which combination of antigens is the best predictor for TFI.

When analysed with the recomScan, the immunoblot results have a higher sensitivity (57.6%) compared to the algorithm outcomes in this study, but at a cost of increase in “false positives.” The algorithm outcome performs better in specificity, PPV, accuracy and clinical utility index.

Setting the algorithm to allow no “false positives” to be included, this still resulted in antigen cut‐offs that would identify 18.2% of all TFI cases in this study with a 100% certainty (CPAF > 9.5 or HSP60 > 3.9; Table 1). When up to a maximum of 30% “false positives” were allowed (cut‐offs: CPAF > 10.7 or OMP2 > 3.9; Table 1), 33.3% of all TFI cases in this study could be identified. However, of all patients that would be considered positive at these antigen cut‐off levels, 21.4% would be “false positive,” that is not have TFI.

As Table 1 shows, all different prediction rules produced by the algorithm include CPAF. TARP was not included in any of the decision rules.

The majority of the TFI cases identified by both the original immunoblot results (as analysed with the recomScan) and the greedy algorithm in this study are the severe TFI cases (either TFI 3 or TFI 4),^17^ for the recomScan 78.9% and 81.8%‐83.3% for the greedy algorithm (Table 2). Most (7/8) of the most severe cases (TFI 4) with occlusions of both fallopian tubes could be identified when the cut‐offs of CPAF > 10.7 or OMP2 > 3.9 were used (Table 2).

**Table 2:** Distribution of severity of TFI in identified TFI patients. TFI classification according to Land et al.^17^ The majority of the TFI cases identified by both the original immunoblot results and the adapted cut‐offs defined in our decision rule are the most severe TFI patients

Cut‐off false positives	TFI identified	TFI2 (%)	TFI3 (%)	TFI4 (%)	Decision rule
0.00	6	1 (16.7)	1 (16.7)	4 (66.7)	CPAF > 9.5 or HSP60 > 3.9
0.05	6	1 (16.7)	1 (16.7)	4 (66.7)	CPAF > 9.5 or HSP60 > 3.9
0.10	6	1 (16.7)	1 (16.7)	4 (66.7)	CPAF > 9.5 or HSP60 > 3.9
0.15	9	2 (22.2)	2 (22.2)	5 (55.6)	CPAF > 8.8 or HSP60 > 3.9
0.20	10	2 (20.0)	2 (20.0)	6 (60.0)	CPAF > 8.8 & MOMP > 8.1, or OMP2 > 4.3
0.25	10	2 (20.0)	2 (20.0)	6 (60.0)	CPAF > 8.8 & MOMP > 8.1, or OMP2 > 4.3
0.30	11	2 (18.2)	2 (18.2)	7 (63.6)	CPAF > 10.7 or OMP2 > 3.9
0.35	11	2 (18.2)	2 (18.2)	7 (63.6)	CPAF > 10.7 or OMP2 > 3.9
0.40	11	2 (18.2)	2 (18.2)	7 (63.6)	CPAF > 10.7 or OMP2 > 3.9
Immunoblot original results	19	4 (4.8)	7 (36.8)	8 (42.1)	
Total TFI	33	11 (33.3)	14 (42.4)	8 (24.2)	

CPAF, chlamydial protease‐like activity factor; HSP60, heat shock protein‐60; MOMP, major outer membrane protein; OMP2, outer membrane protein 2; TFI, tubal factor infertility.

## 4 DISCUSSION

This proof‐of‐principle study researches the contribution of separate types of antibodies in the prediction of TFI, in order to create a predictive value of CAT for TFI. Also, combinations of antibody titres and adjustments in cut‐off values to increase the clinical predictive value of CAT for TFI are studied.

Significant differences between antigen levels in TFI patients and controls were observed, however with these differences no clear distinction could be made between TFI patients and controls (Figure 2), meaning that single antigens cannot be used to identify TFI patients. In this study, we showed that with the adaptation of the original recomScan decision rule, we were able to correctly identify 18% of the TFI patients included in this study, with 100% specificity (Table 1). The adapted decision rule that identifies 18% of TFI patients is “CPAF > 9.5 or HSP60 > 3.9.” The sensitivity of this decision rule can be increased to detect more TFI patients (up to 33%), however this would slightly lower the specificity (100%‐97%), resulting in the inclusion of women without TFI (n = 3; Table 1). In this case, most of the most severe TFI cases (TFI 4) would be included (7 out of 8; Table 2). Due to overlap in titres between the antigens (Figure 2) between TFI patients and controls, we were not able to further distinguish between TFI patients and controls.

Initially, the Mikrogen immunoblot was not developed for the prediction of TFI, but our results show the proof of principle that changing the cut‐off values of the immunoblot can be used to increase identification of TFI patients with a high certainty. This also means that this assay can be used as a regular CAT with the added benefit of being able to predict TFI. Depending on the use of the decision rule in a clinical setting, changes in the decision rule can be made either to optimize specificity or to increase sensitivity. As can be seen in Table 1, with adjustments in the decision rule sensitivity, specificity, negative and positive predictive values change. With the decision rule “CPAF > 9.5 or HSP60 > 3.9,” the sensitivity is relatively limited, but the specificity is high. When implementing such algorithm into the fertility workup, this would mean that the physician could decide to immediately refer these patients to IVF treatment without further testing, reducing the time to IVF and diagnostic costs for these patients.

If such decision rule would be implemented, decisions on how such rule should be used in the fertility workup should be made. The current decision rule identifies a few TFI patients very specifically. This is also reflected in the clinical utility indices. The clinical utility index was developed to incorporate both occurrence of an outcome (eg positive *Chlamydia* serology) and discrimination of the test.^19^ Our decision rule has a high negative clinical utility index of 82.2%‐83.1% which means that is able to accurately exclude most of the TFI‐negative patients.^19^ The positive clinical utility index is low (18.2%‐26.2%) which means that the decision rule is not able to accurately identify all TFI patients, but those that are identified are identified with high certainty. Fertility clinicians will have to choose whether identification of as many TFI patients as possible or identification of fewer TFI patients but with 100% certainty is preferred. When the immunoblot is implemented instead of the current CAT ELISA, then one test would give information on the *Chlamydia* serology (with the manufacturer's algorithm), but will also give additional information on the likeliness of TFI when the cut‐offs from our study are applied to the same antibody measurements. One test would therefore yield more information than the current CAT ELISA.

It should be kept in mind that not all TFI is caused by previous *C* *trachomatis* infections and that this decision rule is based on *C* *trachomatis* serology. Therefore, some TFI patients will not be detected by this decision rule and this decision rule can therefore not fully rule‐out TFI. For patients who are negative with this decision rule, additional diagnostics remain advised. Due to the low sensitivity of the decision rule, it cannot be used as a screening test; however, the high specificity of the decision rule makes it valuable detection of TFI patients when the rule is applied.

The sensitivity, specificity, PPV and NPV of the decision rule are higher than the algorithm of the Mikrogen immunoblot studied previously within the same study population.^15^ This was expected since the decision rule is made on this study population.

We observed that in all decision rules generated with our greedy algorithm, CPAF antibody levels were included, while TARP was not discriminative enough in any of the decision rules.

This is in concordance with the study of Graspeuntner et al^20^ that also found a significant higher level of IgG antibodies targeting chlamydial antigens MOMP, OMP2, CPAF and HSP60, but not TARP in infertile women. Like the findings in our study, their results also highlight the role of HSP60, CPAF and OMP2 in host‐pathogen interactions in females with post‐infectious infertility. The fact that CPAF antibodies were included in all decision rules made with our algorithm shows a strong predictive value for CPAF and may indicate an underlying biological mechanism. A previous trachoma study found that IgG antibody responses to CPAF are likely to be a marker and risk factor for infectious ocular disease severity.^21^ CPAF vaccinations in mice lead to protection against infertility following repeated genital *C* *trachomatis* infections.^22^ CPAF is an important chlamydial virulence factor that enhances persistence of an infection by inhibiting cytokine production of the host's immune system and degradation of host antimicrobial peptides.^23^, ^24^ This suggests that CPAF contributes to *C* *trachomatis* pathogenicity and complications by aiding in ascending of infection.

The decision rule developed in this study is only based on the antigens available in the Mikrogen immunoblot. It shows the proof of principle that the analysis of this existing test can be extended to help improve TFI diagnosis. However, the sensitivity is relatively low for TFI prediction. The antigens in the Mikrogen immunoblot are likely not the only predictive antigens for TFI. The prediction of TFI could be improved with the addition of other antigens. Budrys et al^12^ developed an antigen panel consisting of HSP60, CT376, CT557 and CT443 that distinguish women with TFI from fertile women, who had at least one live birth and normal pelvic findings at laparoscopy, with a detection sensitivity of 63% and a specificity of 100%. However, the same combination of antigens also reacted with sera from STI patients. Even though our decision rule does not have such high sensitivity, we found a high specificity of HSP60 in the detection of TFI. Some antigens used in the study of Budrys et al are not commercially available yet, but combining the antigens found by Budrys et al with the antigens from the immunoblot may improve the sensitivity of the algorithm. Besides bacterial antigens, addition of other clinical variables (eg duration of subfertility), host (genetic) biomarkers^25^ and/or the host vaginal microbiome^26^ may further improve the TFI prediction.

One of the strengths of this study is the well‐defined study population that all underwent tubal patency testing after CAT. The TFI cases have been diagnosed with laparoscopy which is the reference test for detection of TFI.

The Mikrogen immunoblot was developed for the detection of *C* *trachomatis* and not for the detection of TFI specifically. The test was therefore never validated in an infertility population. However, this study shows the proof of principle that by adapting the cut‐off values of the test without further altering the actual test itself, we were able to improve prediction of TFI patients in our study population. Our study population is relatively small and has a high prevalence of TFI and may therefore not be fully representative for a population in a clinical infertility setting. For clinical use, these results should be validated in a larger cohort.

Since the Mikrogen Immunoblot was not developed for the detection of TFI, the use of the statistical terms sensitivity, specificity, negative and positive predictive values is not fully correct in this study; however, the underlying statistical principles remain the same and these terms are familiar to most readers making the interpretation of our outcome easier.

The greedy algorithm used in this study cannot be used in a large data set with a large number of parameters due to its computational complexity which results in very long analysis time. To improve analysis times, heuristics can be developed.

This study is a proof of principle that certain combination antibodies in serum are predictive for TFI and that a commercially available test can be adapted to predict TFI. The decision rule made in this study detects 18% of all TFI patients in our study with a 100% specificity. Validation of this decision rule in a larger cohort is needed before implementing of such model into fertility clinics is possible.

## CONFLICT OF INTEREST

Mikrogen recomLine *C* *trachomatis* immunoblot and recomScan software were provided free of charge by Mikrogen GmbH (Neuried, Germany) and Mediphos Medical Supplies BV (Renkum, The Netherlands).
